# Reno-Duodenal Fistula as a Complication of Staghorn Stones and Renal Pelvis Tumor: A Case Presentation

**DOI:** 10.7759/cureus.60739

**Published:** 2024-05-21

**Authors:** Ziyad A Alnefaie, Aiman E Alsolumany, Faisal H Aljahdali, Omar A Sulaiman, Salih f Aljehani

**Affiliations:** 1 Department of Urology, King Abdulaziz University Hospital, Jeddah, SAU; 2 Department of Urology, King Fahad General Hospital, Jeddah, SAU; 3 College of Medicine, King Abdulaziz University Faculty of Medicine, Rabigh, SAU

**Keywords:** kidney, stone, fistula, retrograde, reno-duodenal fistula, squamous cell carcinoma, staghorn calculi

## Abstract

Reno alimentary fistula, a rare illness characterized by improper connection between the kidney and digestive tract, can lead to urinary tract infections, abscesses, and severe sepsis. It can also be caused by various factors such as chronic infections, malignancy, cryoablation, or abdominal surgical procedures.

We present a case of a 60-year-old man with bilateral staghorn stones who was diagnosed with reno-duodenal fistula and underwent a right simple nephrectomy and fistula closure. The histopathology revealed a well-differentiated squamous cell carcinoma that originated from the renal pelvis.

## Introduction

Reno-alimentary fistula is a rare condition characterized by an abnormal connection between the kidney and the digestive tract. This leads to the flow of urine into the gastrointestinal system or the reverse flow of digestive fluids into the urinary system [1,2].

Duodenal fistulas are uncommon and can develop in various clinical scenarios. They can develop in a notable proportion of patients who have undergone surgical repair for perforated peptic ulcers, with a reported occurrence ranging from 2% to 7%. Moreover, they are present in approximately 4% of cases of severe acute pancreatitis, 3% after gastrectomy for gastric cancer, and 1% following the diagnostic procedure called endoscopic retrograde cholangiopancreatography (ERCP) [3-6].

Reno-duodenal fistula is a rare event that typically develops due to injury or inflammation. One significant complication of reno-duodenal fistula is urinary tract infection, as the introduction of digestive contents introduces harmful bacteria, leading to infections. Symptoms of these infections may include discomfort, pain, fever, and frequent urination [7].

When it comes to patients with duodenal fistulas, surgical intervention, such as duodenal repair or resection, can be an effective management approach, particularly when performed by an experienced surgeon [8].

## Case presentation

**Figure 1:**
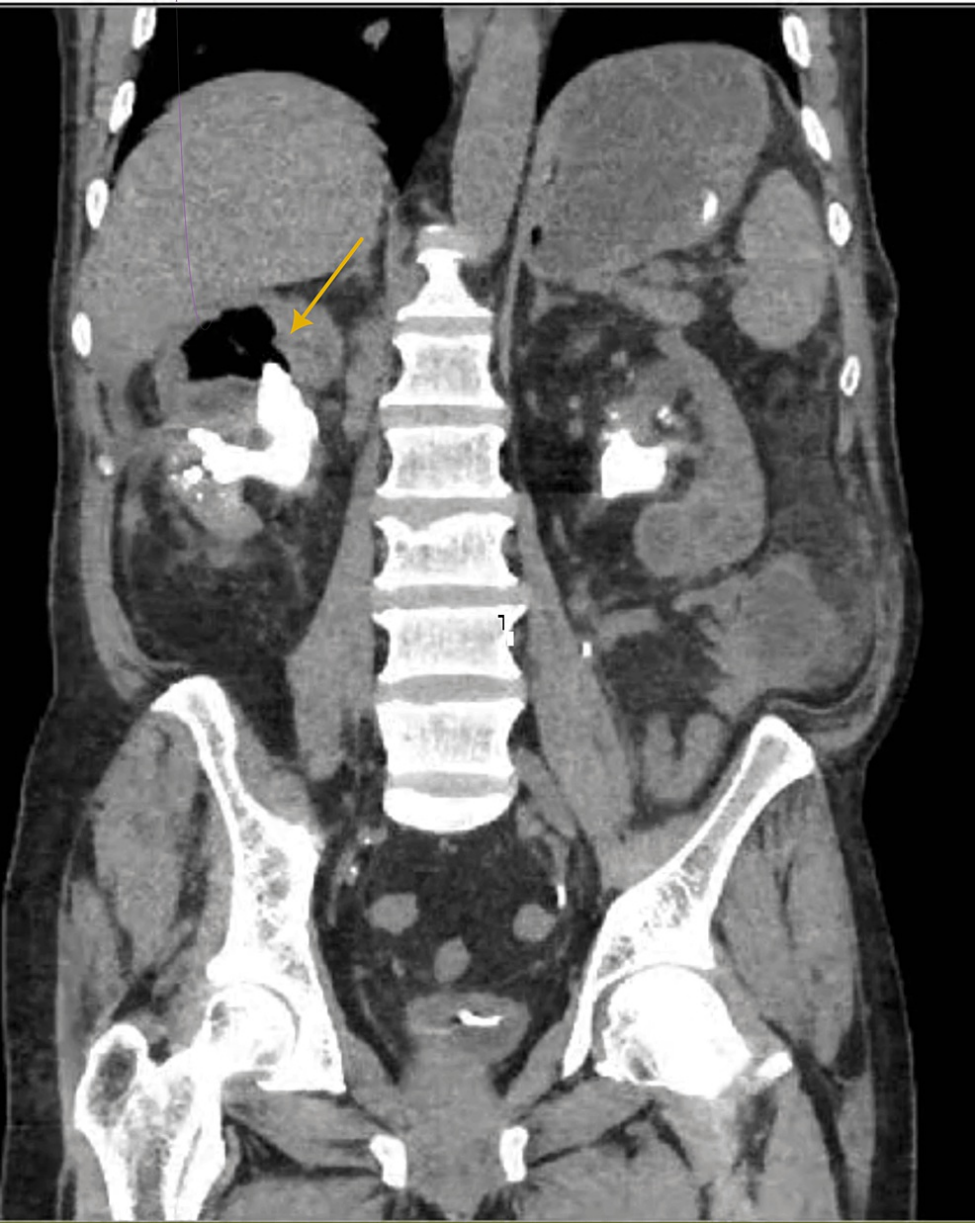
Coronal plane CT KUB show the presence of bilateral staghorn stones and right emphysematous pyelitis

**Figure 2:**
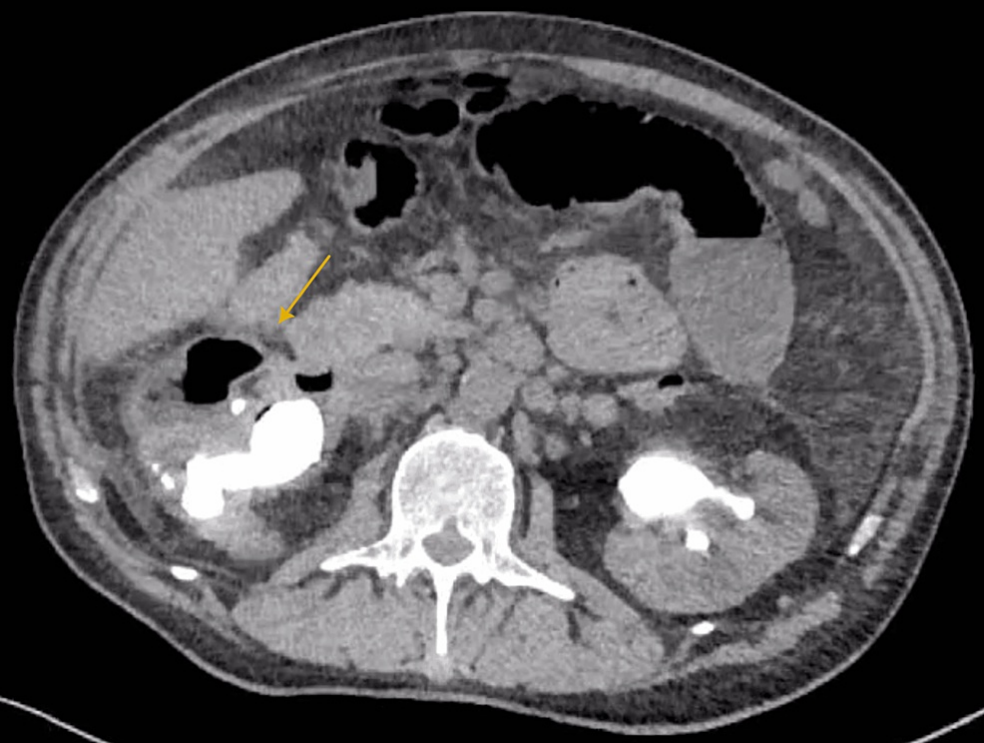
Axial plane CT KUB show the presence of bilateral staghorn stones and right emphysematous pyelitis

This article presents the case of a 60-year-old male patient who was diagnosed with bilateral staghorn stones (Figures 1, 2) and was managed by bilateral placement of DJ stents. Three months later, he presented to the emergency department at King Fahad General Hospital with complaints of abdominal pain associated with nausea, vomiting, and constipation. The patient was conscious, oriented, and vitally stable during the examination. The abdominal examination was unremarkable, and no flank tenderness was observed. The laboratory results showed leukocytosis, CT scan confirmed the presence of right emphysematous pyelitis and dilated small bowel.

**Figure 3:**
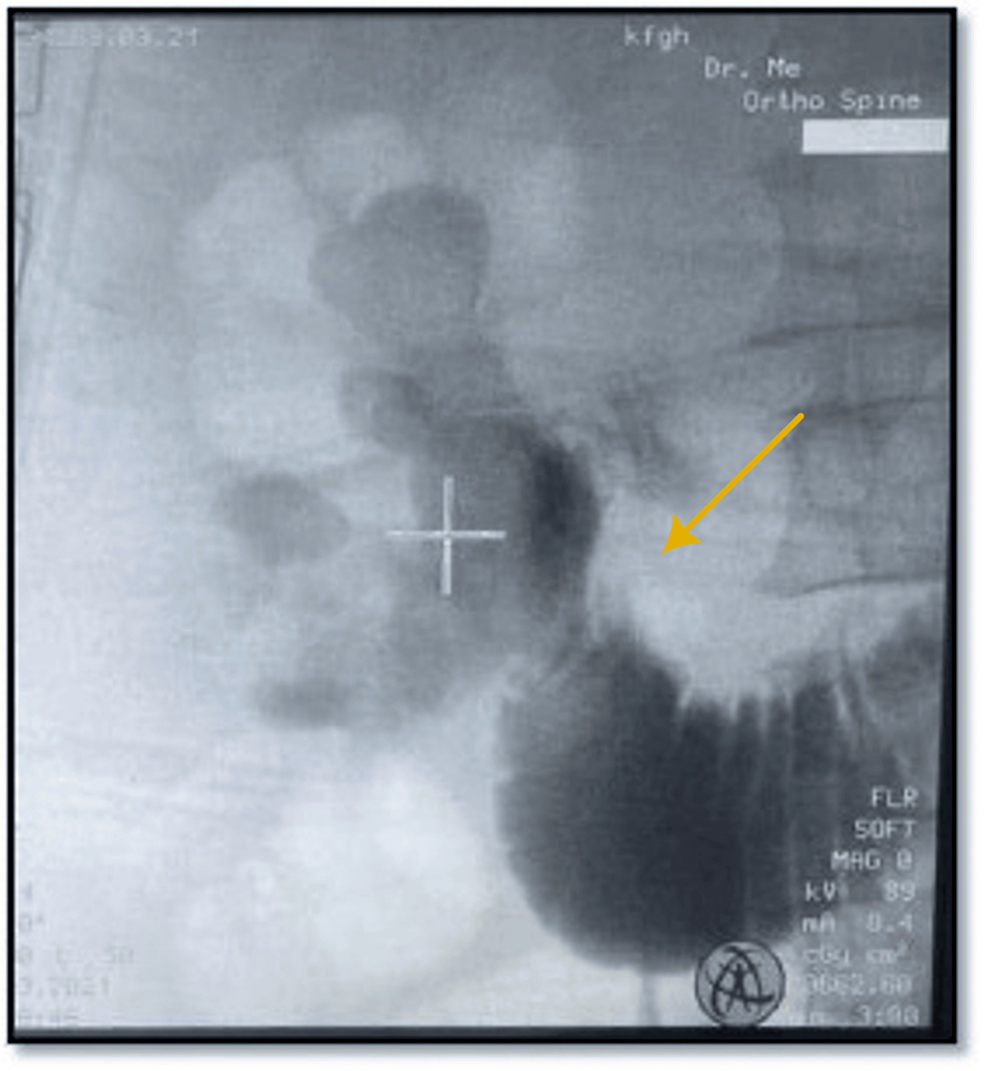
Right retrograde pyelography showing contrast in pelvicalyceal system and duodenum

**Figure 4:**
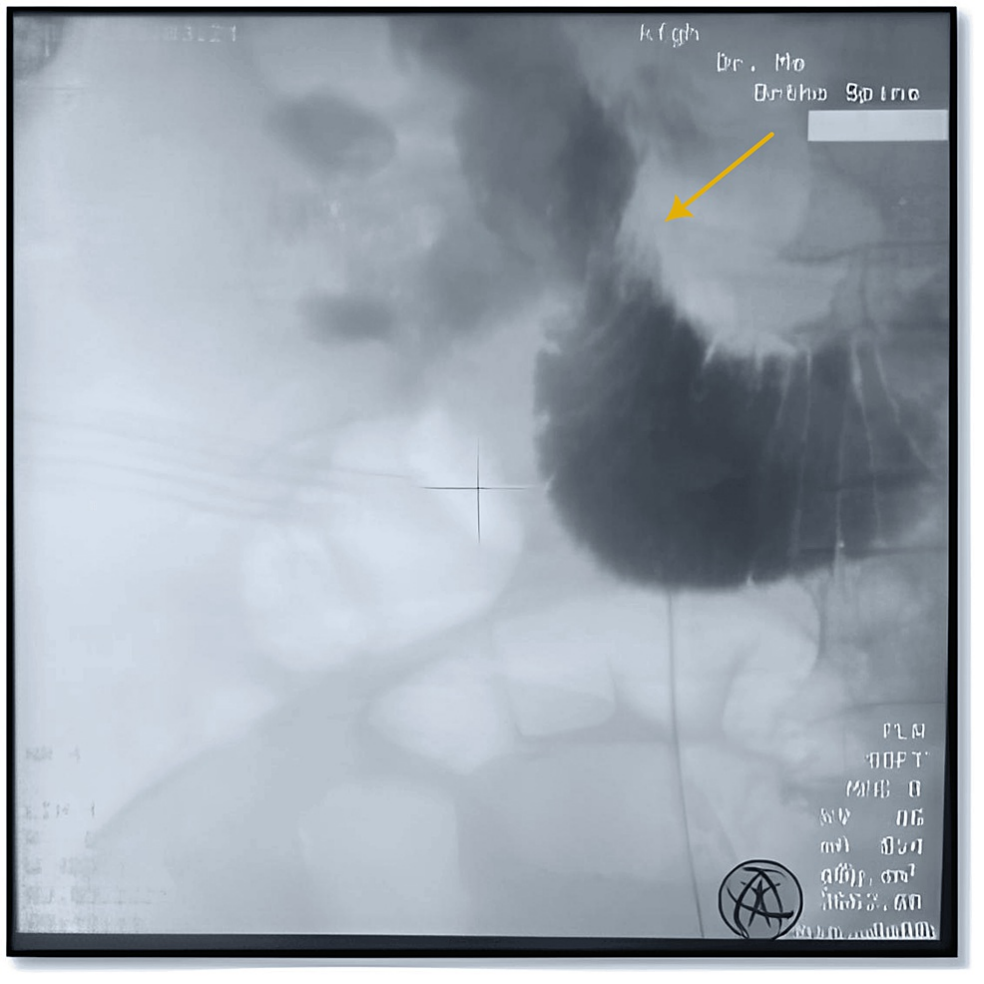
Right retrograde pyelography showing contrast in pelvicalyceal system and duodenum

The patient was admitted as a case of emphysematous pyelitis. The management plan included administering broad-spectrum antibiotics and providing supportive care. After one week of antibiotic (ceftriaxone), bilateral DJ stent exchange was planned, while right retrograde pyelography was being conducted showed contrast draining from the pelvicalyceal system to duodenum (Figures 3, 4), suggesting right reno-duodenal fistula, . A right nephrostomy tube was inserted for maximum urine drainage. Further investigations involved upper gastrointestinal endoscopy, which confirmed the diagnosis of reno-duodenal fistula.

A collaborative approach was undertaken, which involved both the urology and general surgery teams to address the condition. The patient underwent a right simple nephrectomy and primary fistula repair.

Postoperative care included close monitoring for complications, pain management, wound assessments, and renal function monitoring. Postoperative day four the patient signed DAMA due to financial issues and left the hospital prematurely.

The histopathology report showed a well-differentiated squamous cell carcinoma that originated from the renal pelvis and has metastasized to the paracaval lymph node. Subsequent investigations indicated extensive disease involving the duodenum, peritoneum, liver, small bowel, lymph nodes, and suspected involvement of the inferior vena cava.

Despite the interruption in postoperative management and the advanced stage of the disease, the initial treatment plan aimed to address the complications, eliminate infection, manage the malignancy, and stabilize the patient's condition.

## Discussion

A rare phenomenon is the occurrence of a fistula that connects the upper urinary tract and the digestive tract. Among the different types of fistulas that establish a connection between the urinary and intestinal tracts, reno-alimentary fistulas account for less than 1% of cases. Several underlying pathological processes can give rise to the development of such fistulas [2,9].

The primary cause of reno-alimentary fistulas is often iatrogenic, meaning they are inadvertently caused by interventional procedures [10]. One common example is the formation of reno-alimentary fistulas due to procedures like percutaneous nephrostomy [11]. Nevertheless, it is crucial to acknowledge that other factors can contribute to the development of these fistulas.

The presence of a reno-alimentary fistula not only increases the risk of urinary tract infections but also promotes abscess formation due to the interaction between urine and digestive contents, creating an environment suitable for bacterial growth. Abscesses result in significant pain, swelling, and general illness [9,12]. Furthermore, severe cases of reno-alimentary fistulas can lead to life-threatening sepsis, characterized by systemic infection, causing widespread inflammation, organ dysfunction, and potential fatality if untreated [13]. 

A timely and accurate diagnosis of a reno-alimentary fistula, particularly a reno-duodenal one, is vital due to its potential impact on the patient's well-being [9]. A comprehensive investigation is necessary to determine the optimal approach for managing the fistula and its related complications [14].

In this case, the development of the reno-duodenal fistula is attributed to the presence of the staghorn stone. Previous studies have also identified iatrogenic complications, specifically those resulting from nephrostomy and percutaneous nephrolithotomy, as potential causes of reno-duodenal fistula formation. However, thus far, there have been no reported cases in the available literature of a reno-duodenal fistula arising solely from the chronic irritation caused by stones. 

In our case intraoperative finding, we encountered significant adhesions between the kidney and the duodenum. Given the high risk of recurrence associated with such findings, we decided to perform radical nephrectomy with fisulectomy. 

## Conclusions

In summary, a reno-alimentary fistula, including a reno-duodenal fistula, is an uncommon condition characterized by an abnormal connection between the kidney and the digestive tract. It can give rise to various complications, such as urinary tract infections, the formation of abscesses, sepsis, kidney damage, and impaired kidney function. Diagnosing the condition promptly and accurately is crucial to ensuring appropriate management. Diagnostic methods such as retrograde pyelography and upper endoscopy are helpful to identify these fistulas. Managing the fistula and its associated complications requires a multidisciplinary approach.
